# Perceived Impact of Digital Health Maturity on Patient Experience, Population Health, Health Care Costs, and Provider Experience: Mixed Methods Case Study

**DOI:** 10.2196/45868

**Published:** 2023-07-18

**Authors:** Leanna Woods, Ronald Dendere, Rebekah Eden, Brittany Grantham, Jenna Krivit, Andrew Pearce, Keith McNeil, Damian Green, Clair Sullivan

**Affiliations:** 1 Centre for Health Services Research Faculty of Medicine The University of Queensland Herston Australia; 2 Queensland Digital Health Centre Faculty of Medicine The University of Queensland Herston Australia; 3 Digital Health Cooperative Research Centre Sydney Australia; 4 UQ Business School The University of Queensland Brisbane Australia; 5 Healthcare Information and Management Systems Society Singapore Singapore; 6 eHealth Queensland Brisbane Australia; 7 Metro North Hospital and Health Service Brisbane Australia

**Keywords:** digital health, health information systems, digital maturity, digital hospital, evaluation study, impact, outcome assessment, qualitative research, health services research

## Abstract

**Background:**

Health care organizations understand the importance of new technology implementations; however, the best strategy for implementing successful digital transformations is often unclear. Digital health maturity assessments allow providers to understand the progress made toward technology-enhanced health service delivery. Existing models have been criticized for their lack of depth and breadth because of their technology focus and neglect of meaningful outcomes.

**Objective:**

We aimed to examine the perceived impacts of digital health reported by health care staff employed in health care organizations across a spectrum of digital health maturity.

**Methods:**

A mixed methods case study was conducted. The digital health maturity of public health care systems (n=16) in Queensland, Australia, was examined using the quantitative Digital Health Indicator (DHI) self-assessment survey. The lower and upper quartiles of DHI scores were calculated and used to stratify sites into 3 groups. Using qualitative methods, health care staff (n=154) participated in interviews and focus groups. Transcripts were analyzed assisted by automated text-mining software. Impacts were grouped according to the digital maturity of the health care worker’s facility and mapped to the quadruple aims of health care: improved patient experience, improved population health, reduced health care cost, and enhanced provider experience.

**Results:**

DHI scores ranged between 78 and 193 for the 16 health care systems. Health care systems in the high-maturity category (n=4, 25%) had a DHI score of ≥166.75 (the upper quartile); low-maturity sites (n=4, 25%) had a DHI score of ≤116.75 (the lower quartile); and intermediate-maturity sites (n=8, 50%) had a DHI score ranging from 116.75 to 166.75 (IQR). Overall, 18 perceived impacts were identified. Generally, a greater number of positive impacts were reported in health care systems of higher digital health maturity. For patient experiences, higher maturity was associated with maintaining a patient health record and tracking patient experience data, while telehealth enabled access and flexibility across all digital health maturity categories. For population health, patient journey tracking and clinical risk mitigation were reported as positive impacts at higher-maturity sites, and telehealth enabled health care access and efficiencies across all maturity categories. Limited interoperability and organizational factors (eg, strategy, policy, and vision) were universally negative impacts affecting health service delivery. For health care costs, the resource burden of ongoing investments in digital health and a sustainable skilled workforce was reported. For provider experiences, the negative impacts of poor usability and change fatigue were universal, while network and infrastructure issues were negative impacts at low-maturity sites.

**Conclusions:**

This is one of the first studies to show differences in the perceived impacts of digital maturity of health care systems at scale. Higher digital health maturity was associated with more positive reported impacts, most notably in achieving outcomes for the population health aim.

## Introduction

### Background

Globally, health care is undergoing a rapid digital transformation [1,2]. The Global Strategy for Digital Health developed by the World Health Organization aims to improve global health by accelerating the development and adoption of digital health solutions that will enable countries to use health data to promote health and well-being [1].

Many organizations understand that they need to implement new technologies; however, the optimal pathway to digital excellence is often unclear [3], and so health care providers are creating their own digital health strategies [4]. These strategies require a baseline understanding of the current state of the digital health of the organization before planning future implementation and transformation [5]. Such evaluations are usually achieved via the assessment of digital health maturity [6]. National digital health strategies outline the need for health services to measure and improve their digital maturity or digital capability [7]. Digital health maturity assessments allow health care organizations to understand their readiness for integrating digital technologies and the development of roadmaps for improving patient care [5]. For example, the Electronic Medical Record Adoption Model scores hospitals in various countries to track the aspirational outcomes of digital transformations [8]. However, selecting the most appropriate digital health maturity model for the right health care context remains challenging [9], and existing models have often been criticized for lacking depth and breadth because of their focus on technology while neglecting meaningful consumer, patient, and system outcomes [10-12]. Focusing on the depth of technology implementation, instead of health care system outcomes achieved through technology, is now an outdated approach, and there are calls for a more balanced, outcome-focused perspective [13]. As digital maturity captures aspects of business processes, organizational characteristics, information, and people [14] (not simply the presence or absence of a particular technological intervention), it is increasingly used as a model for benchmarking within and between health care providers [11]. This ensures that the downstream impacts of technology implementations on a connected digital health care system, such as artificial intelligence and precision medicine, are captured in evaluations [13].

Despite the shift to outcome-focused digital maturity models, evaluations have so far failed to reliably investigate the impact of advancing maturity on the quadruple aims of health care [10]. The concept of quadruple aims of health care [15] is now accepted as a holistic framework for assessing the various outcomes of digital health [13,16]. Evolving from the 2008 Institute of Healthcare Improvement “triple aim” [17], which consists of enhancing patient experience, improving population health, and reducing the cost of care, the quadruple aims of health care also include considerations of the provider experience. Consistent with health care intervention evaluations—for example, in workforce [18,19], innovation [20], and the COVID-19–pandemic [21] contexts—digital transformation should be evaluated in the context of the effect it has on patient experience, population health, health care costs, and the provider (clinician) experience [13].

### Objective

This gap in the literature prompted the research question: *How do the outcomes of digital transformation as mapped to the quadruple aim differ according to the digital health maturity of the health care system?* Our objective was to examine the perceived impacts of digital health reported by health care staff employed at sites across a spectrum of digital health maturity.

## Methods

### Study Design

A mixed methods case study design was used in this research. Data were collected between December 2020 and July 2021 via quantitative surveys, qualitative interviews, and focus groups [22].

### Case Setting

The study was conducted at Queensland Health, the public health service in the Australian state of Queensland, which delivers universal health care to >5 million people across 16 geographically defined health care systems. These health care systems provide a variety of health care services ranging from small rural multipurpose health care clinics to quaternary academic hospitals [23,24]. Each health care system supports inpatient and outpatient services, including emergency care, hospital care, primary health care, and allied health care [25]. Of the 16 health care systems, 9 (56%) have at least 1 health care service with an electronic medical record (EMR) implemented based on a single-instance Cerner integrated EMR. Other health care services (7/16, 44%) use a combination of disparate systems and paper-based records to track the patient journey.

### Data Collection and Data Analysis

#### Quantitative Survey: Digital Health Maturity Assessment

To determine the digital health maturity of each of the 16 health care systems, the Digital Health Indicator (DHI) self-assessment survey was used. The DHI, developed by the Healthcare Information and Management Systems Society (HIMSS), consists of 121 items measured on a 5-point Likert scale ranging from “not enabled” to “fully enabled,” representing the digital health capability across the following 4 dimensions [4,26]:

- Interoperability: the digital infrastructure strategy that makes data more accessible, secure, and sharable among stakeholders
- Person-enabled health: health care services that support the needs and unique life circumstances of individuals and populations to manage their health and wellness
- Predictive analytics: transformation of data into information, knowledge, and insights to create real-world evidence to inform decisions
- Governance and workforce: the vision and system-level strategy to guide digital health implementations

Survey participants were identified through performing presentations and eliciting recommendations at a variety of state-level forums and departments including the Health Services Chief Executive Forum, the Chief Information Officer Forum, eHealth Queensland, and Clinical Excellence Queensland to identify the most senior digital health subject matter expert within a given health care system (eg, chief information officer and director of digital health). Upon receiving the recommendations, individual emails were sent to prospective participants, with a participant information form attached and a follow-up email a week later. Once the completed survey was returned, the DHI score was computed using a proprietary algorithm, with a maximum DHI score of 400 denoting optimal maturity. We calculated the lower and upper quartiles of the DHI scores, which we subsequently used to cluster the sites into 3 groups. Upon inspecting the DHI quartiles, we were satisfied that they sufficiently stratified the sites to allow us to explore the impacts of digital health based on digital maturity (DHI scores). The first group (which we designated the “low digital health maturity” group) consisted of sites with DHI scores below the lower quartile; the “intermediate digital health maturity” group consisted of sites with DHI scores between the lower and upper quartiles; and the “high digital health maturity” group consisted of sites with DHI scores above the upper quartile.

#### Qualitative Interviews and Focus Groups: Outcomes of Digital Health Maturity

##### Overview

The qualitative data collection occurred concurrently with the quantitative data collection. Purposeful sampling of health care staff was used to capture a range of perspectives to account for diverse workforce roles (ie, clinicians, directors, executives, and patient engagement leaders). Participants were identified via the state-wide health executive forum and invited to participate if they had an understanding of and responsibility for health care knowledge management in their department [27]. To ensure role representation at each site, site contacts were used to identify suitable additional participants. Participation in this research was voluntary, and in line with the ethical principles, potential participants were provided with detailed participant information and consent forms, which they signed before partaking in the interview.

The interview protocol was developed and tested by the HIMSS in previous digital health capability assessments in other jurisdictions [28]. The semistructured interview guide contained questions pertaining to strategic vision, experiences of implementations, and evaluations of digital transformations, and the questions were targeted toward dimensions of digital maturity (interoperability, person-enabled health, predictive analytics, and workforce and governance). The interview guide was tailored to each professional group and used in each qualitative interview and focus group. Focus groups were offered when ≥2 participants had the same role in the same setting (eg, nursing).

Interviews and focus groups were conducted via web-based videoconferencing using the camera function to ensure that nonverbal body language could be considered. A total of 2 interviewers were present. Interviewer 1 was a HIMSS analyst external to the setting with extensive health care experience in consulting and clinical projects. Interviewer 2 was a researcher or research assistant who managed the recording, asked follow-up questions for clarification, and ensured appropriate management of biases and assumptions. Senior researchers (RE and CS) with experience in conducting digital health research at the study setting mentored interviewers before and during data collection to ensure that the interviews were rigorously conducted. All interviews and focus groups were audio recorded, deidentified using a unique identifier code, manually transcribed verbatim by a transcription service, and verified by a researcher (LW). Participants were given the opportunity to review their transcripts before conducting the analysis. Data collection concluded for each site when perceptions from a diverse range of workforce roles had been collected, with no additional insights emerging.

Qualitative data analysis was conducted in 2 stages.

##### Stage 1: Semiautomated Text Analysis

To assist the inductive analysis of the large data set, we applied a validated [29] and increasingly adopted [30] text-mining software, Leximancer (version 4.5; Leximancer Pty Ltd), to identify the concepts in the interview data. Leximancer uses artificial intelligence to identify novel linkages and groupings of specific terminology and automatically codes 2-sentence segments of text using an inbuilt thesaurus [31]. In total, 3 researchers (BG, RD, and LW) used Leximancer to analyze the concepts discussed by interview participants within each digital maturity group (ie, high digital maturity, intermediate digital maturity, and low digital maturity) and consistently applied the following steps [31]:

1. *Formatting transcripts:* each anonymized transcript was reviewed to become familiar with the content, screened for grammatical or formatting errors, and formatted consistently.
2. *Text processing and concept seed generation:* after running an initial analysis for each group to understand the concepts generated, redundant conversational words were added to the stop list before text processing (“name,” “obviously,” “stuff,” “yeah,” “probably,” and “things”). All other default settings of the Leximancer software remained unchanged.
3. *Concept editing:* only automatically defined concepts were used, and thesaurus settings were unchanged from default.
4. *Concept coding:* all software settings were unchanged from default.
  Outputs from the Leximancer analysis are visual concept maps where the most frequently connected concepts are presented within a colored circle and related concepts are presented as gray dots. This provided a broad view and patterns in the qualitative data set.

##### Stage 2: Thematic Analysis

To obtain a rich understanding of the stage-1 results, a researcher-led thematic analysis was conducted following a 6-step method [32]. First, 3 researchers (LW, RD, and BG) familiarized themselves with the data by reading and rereading interview transcripts and recorded initial ideas and thoughts. Second, researchers independently and systematically reviewed Leximancer-generated concepts (stage 1) in the individual digital maturity groups (BG reviewed group-1 data, LW reviewed group 2, and RD reviewed group 3). Third, in search of themes, a series of “queries” in Leximancer were conducted as potential lines of inquiry. The top 5 Leximancer-identified concepts (eg, patients) and the relationship with the top 3 to 5 co-occurring concepts (eg, patients and systems) were examined, generating a concept group. The researchers independently performed text extraction, generating a preliminary interpretation of the meaning of each concept group. Interrater reliability assessments were performed on a subset of the data, and consensus was reached among the independent reviewers. Fourth, working within each digital maturity group, researchers collectively reviewed preliminary interpretations, discussed theoretical assumptions, and refined them by grouping similar interpretations in a table using word-processing software to generate themes. Fifth, themes were defined as impacts and named. The quadruple aims of health care (ie, patient experience, population health, health care costs, and provider experience) [15] guided the categorization of perceived impacts. The researchers collaboratively categorized impacts into the quadruple aims. Definitions for each of the quadruple aims were used to identify concepts significant to the patient (ie, preferences, satisfaction, communication, access, engagement, and use); population (ie, equity, access, disparities, and health outcomes); health care system (ie, costs and use); and providers (ie, satisfaction, workload, and preferences) [33]. Researchers generated insights by analyzing findings across the aims and classified the impacts as positive, negative, or mixed in sentiment; in some cases, the impact was not mentioned by participants. Consensus was achieved through group discussions and the review of text segments as necessary. The report was then generated.

The steps taken to improve the reliability of the results included member checking [34] of transcripts (by individual staff as requested on their participant consent form) and digital maturity reports (by site contacts), the socialization of key research findings (with state-wide project governance group members), and presentations (with attendees at internal Queensland Health forums). No adjustments were made to the analysis or results during any of these member-checking events beyond the clarification of acronyms, software, and context-specific terminology.

### Ethics Approval

The study was conducted in accordance with the National Statement on Ethical Conduct in Human Research [35] and received multisite ethics approval from Metro North Hospital and Health Service (project HREC/RBWH/88695), the University of Queensland, and Queensland University of Technology.

## Results

### Participant Demographics and Health Care System Maturity

In total, 154 individuals (Table 1) participated in 134 interviews and 9 focus groups (Table 2).

**Table 1:** Participant demographics of health care staff involved in interviews and focus groups (n=154).

Role	Participants, n (%)
Clinicians (eg, allied health, pharmacists, nurses, and physicians)	65 (42.2)
Executive (eg, chief information officer, and chief transformation officer)	26 (16.9)
Clinical manager (eg, director of allied health, director of nursing, clinical nurse consultant, chief nursing officer, and general manager primary care)	21 (13.6)
Patient engagement leader (eg, consumer engagement officer)	21 (13.6)
Director (eg, director of sustainability, executive finance or digital, director medical services, director of digital health, and executive nursing services)	10 (6.5)
Health information managers and informatics team members	9 (5.8)
Aboriginal and Torres Strait Islander engagement team members	2 (1.3)

**Table 2:** Interviews and focus groups per site.

Site	Interviews (n=134), n (%)	Focus groups (n=9), n (%)	Participants (n=154), n (%)
1	7 (5.2)	0 (0)	7 (4.5)
2	9 (6.7)	0 (0)	9 (5.8)
3	10 (7.5)	0 (0)	10 (6.5)
4	7 (5.2)	1 (11.1)	9 (5.8)
5	10 (7.5)	0 (0)	10 (6.5)
6	9 (6.7)	0 (0)	9 (5.8)
7	9 (6.7)	0 (0)	9 (5.8)
8	5 (3.7)	0 (0)	5 (3.2)
9	7 (5.2)	3 (33.3)	15 (9.7)
10	8 (6)	1 (11.1)	10 (6.5)
11	6 (4.5)	2 (22.2)	10 (6.5)
12	8 (6)	0 (0)	8 (5.2)
13	12 (9)	0 (0)	12 (7.8)
14	9 (6.7)	0 (0)	9 (5.8)
15	7 (5.2)	0 (0)	7 (4.5)
16	11 (8.2)	2 (22.2)	15 (9.7)

The DHI scores of health care systems in Queensland Health (n=16; mean 143, SD 35.3; range 78-193) are reported elsewhere [28]. In this study, we grouped the sites using the lower (116.75) and upper (166.75) quartiles of the DHI scores. The DHI score of the health care system in which the participants worked was used to categorize the perceived impacts of digital transformations in high, intermediate, and low digital health maturity sites (Table 3). Multimedia Appendix 1 summarizes the DHI and dimension level (ie, interoperability, person-enabled health, predictive analytics, and governance and workforce) scores for high, intermediate, and low digital health maturity sites.

**Table 3:** Digital maturity categories of health care systems (n=16).

Digital maturity category	DHI^a^ score	Sites, n (%)	Site ID
High	≥166.75	4 (25)	4, 5, 10, and 11
Intermediate	116.75<DHI<166.75	8 (50)	3, 6, 8, 9, 12, 13, 14, and 16
Low	≤116.75	4 (25)	1, 2, 7, and 15

^a^DHI: Digital Health Indicator.

### Stage 1: Semiautomated Text Analysis

**Figure 1:**
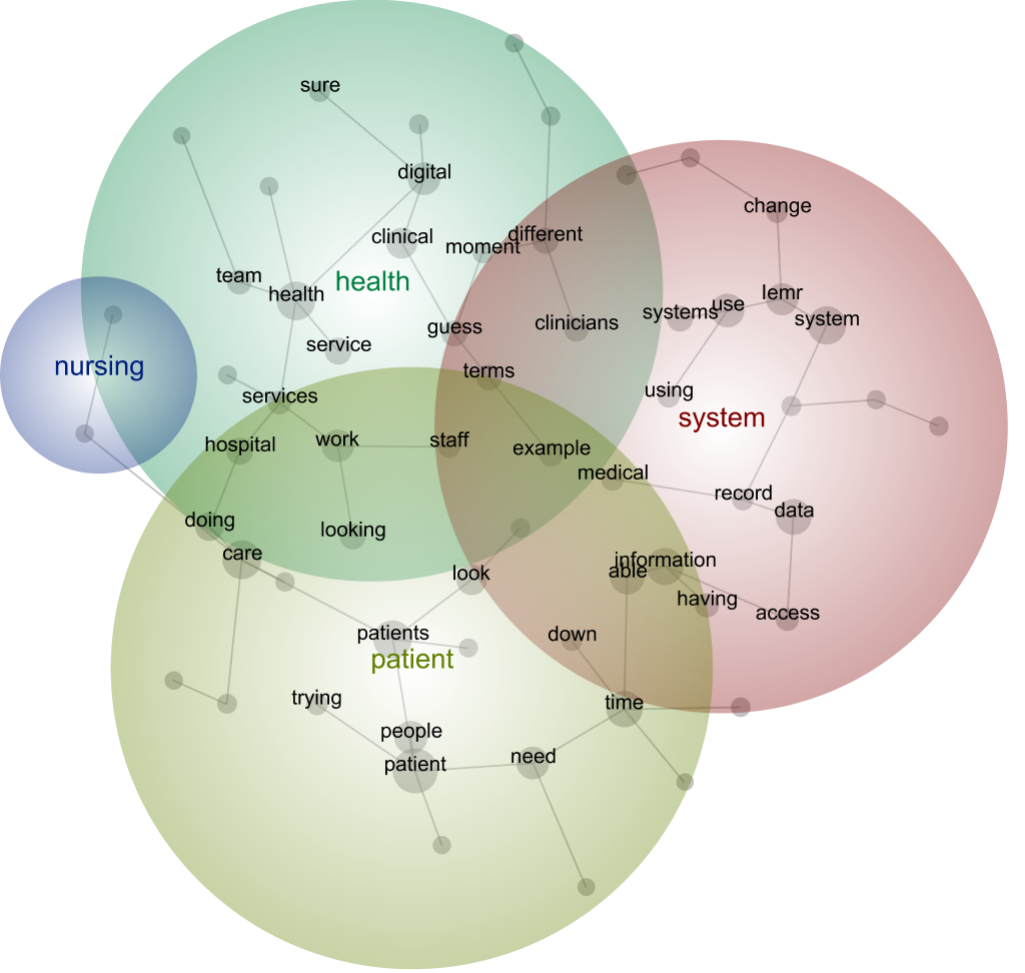
Leximancer output of high digital maturity interview data. iEMR: integrated electronic medical record.

**Figure 2:**
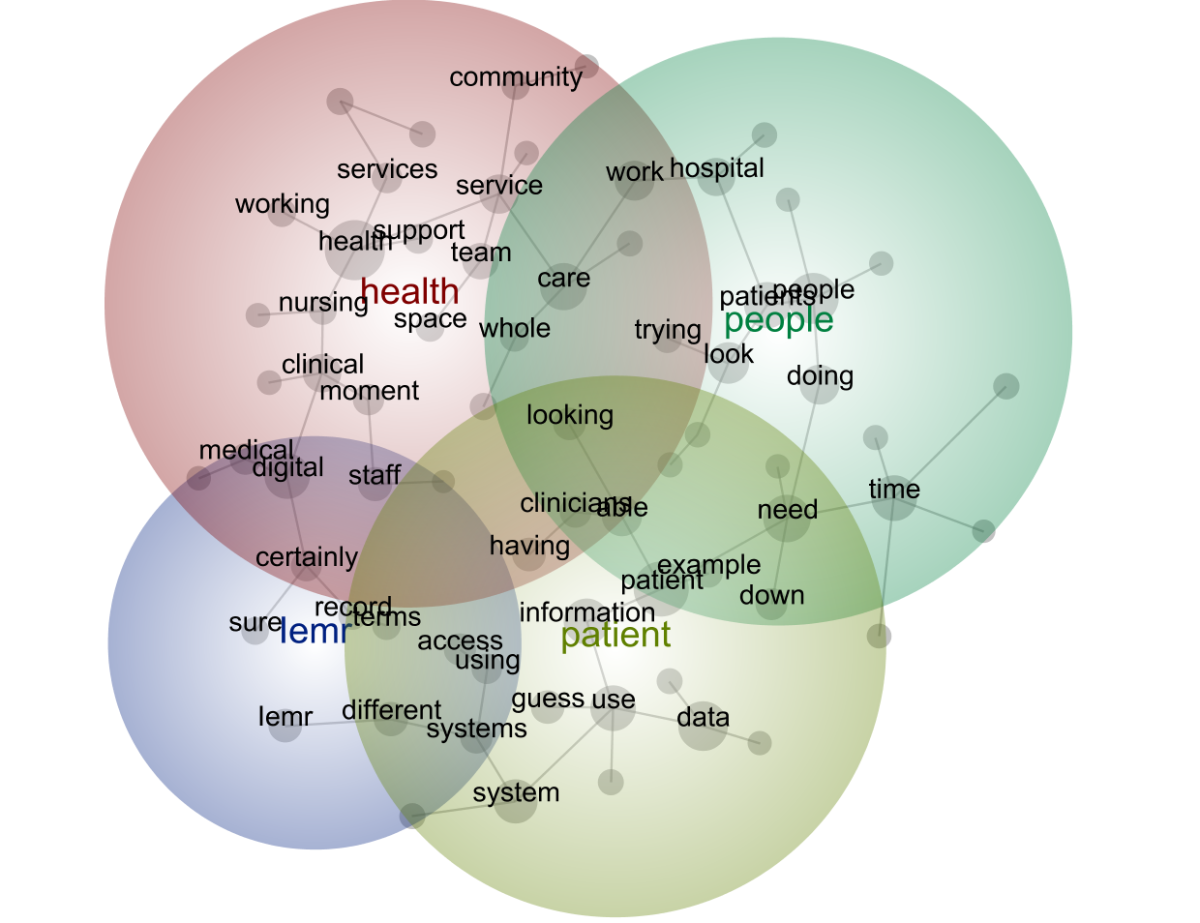
Leximancer output of intermediate digital maturity interview data. iEMR: integrated electronic medical record.

**Figure 3:**
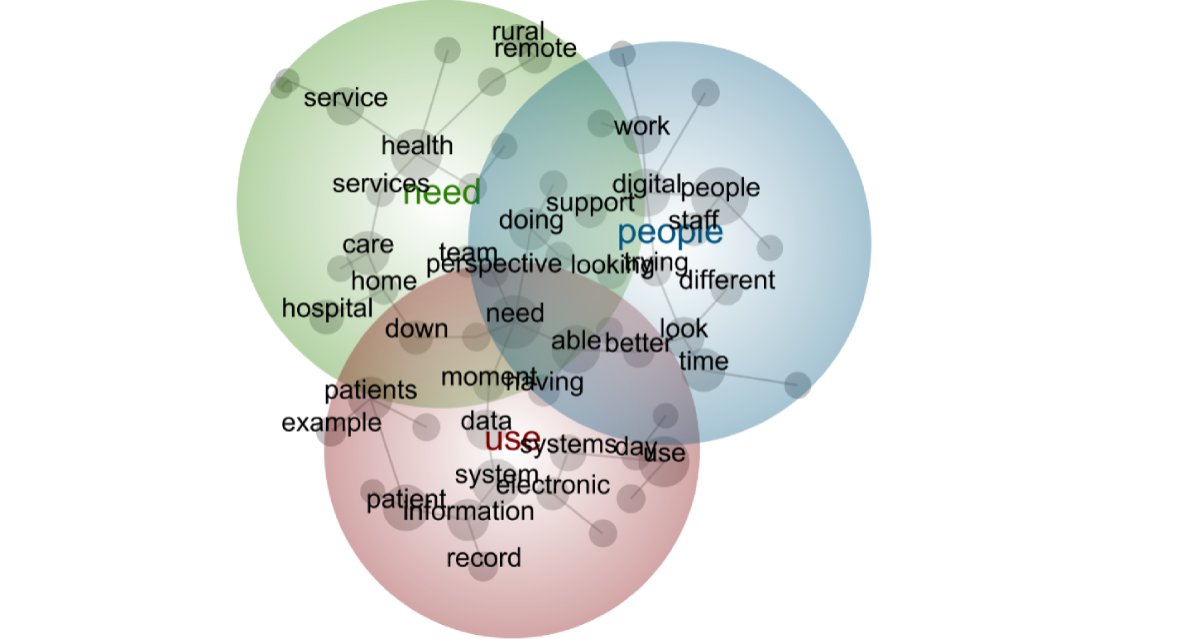
Leximancer output of low digital maturity interview data.

The first stage provides a visual depiction of the outputs from the Leximancer analysis for each digital maturity group. High digital maturity health care systems discussed the following 4 key concepts (in order of prevalence): system (most prevalent), patient, health, and nursing (least prevalent; Figure 1). For intermediate digital maturity health care systems, the 4 most prevalent concepts were health, patient, people, and the integrated EMR (Figure 2). For low digital maturity health care systems, the most prevalent concepts were use, need, and people (Figure 3). The bubbles are heat mapped to represent the concept frequency (where red depicts the most prevalent concepts, followed by orange, green, and blue). The top 25 most frequently occurred concepts are presented in Multimedia Appendix 2.

### Stage 2: Thematic Analysis

#### Overview

Overall, 18 impacts were identified and mapped to the quadruple aims of health care (Textbox 1).

The impacts varied per digital maturity category (ie, high, intermediate, and low digital health maturity) in terms of presence and sentiment (Table 4). Multimedia Appendix 3 presents representative quotes for each maturity group and impact.

**Perceived impacts of digital transformation mapped to the quadruple aims of health care.**

**Patient experience**

- Telehealth for health care access and flexibility
- Patient-provider communication
- Patient digital literacy
- Patient experience data tracking
- Find and maintain patient health record

**Population health**

- Telehealth for health care access and efficiency
- Organization factors (eg, strategy, policy, and vision) for health service delivery
- Interoperability between systems
- Tracking patient journey
- Clinical risk mitigation

**Health care costs**

- Resource burden of ongoing investments
- Resource burden of a skilled workforce
- Economic benefit visible

**Provider experience**

- Usability
- Change fatigue
- Acceptance
- Management of clinician workloads
- Network and infrastructure impacts

**Table 4:** Perceived quadruple aim impacts reported by staff in health care systems classified as high, intermediate, and low digital health maturity.

Quadruple aim of health care impact		High digital health maturity sites	Intermediate digital health maturity sites	Low digital health maturity sites
**Patient experience**				
	Telehealth for health care access and flexibility	+^a^	+	+
	Patient-provider communication	−^b^	−	NM^c^
	Patient digital literacy	NM	−	+/−^d^
	Patient experience data tracking	+	−	NM
	Find and maintain patient health record	NM	+/−	−
**Population health**				
	Telehealth for health care access and efficiency	+	+	+
	Organization factors (eg, strategy, policy, and vision) for health service delivery	−	−	−
	Interoperability between systems	−	−	−
	Tracking patient journey	+	+/−	−
	Clinical risk mitigation	+	+	NM
**Health care costs**				
	Resource burden of ongoing investments	+/−	−	−
	Resource burden of a skilled workforce	+/−	−	−
	Economic benefit visible	NM	−	NM
**Provider experience**				
	Usability	+/−	−	−
	Change fatigue	−	−	−
	Acceptance	NM	+/−	+/−
	Management of clinician workloads	NM	+	NM
	Network and infrastructure impacts	NM	NM	−

^a^+: positive sentiment.

^b^−: negative sentiment.

^c^NM: not mentioned.

^d^+/−: mixed sentiment.

#### High Digital Health Maturity

At sites with high digital health maturity, telehealth was identified as an enabler to improve access to care and flexibility for patients, positively contributing to the patient experience. The flexibility of telehealth alleviates some of the logistics-related stress associated with face-to-face health care delivery, “When it means they don’t have to get in their car and drive for such long distances, or get on a plane, and stay overnight...” (A010). Increased use of computers in high digital maturity sites was seen as a barrier to face-to-face patient-provider communication:

…patients [feel] that the attention of the nurse or the doctor or the allied health professional, is on the computer, rather than on the patient.

F009 and F010

However, with more data about patient experience, providers could adjust health care in meaningful ways for patients, positively contributing to patient experience data tracking:

We’re starting to play with this idea of if we can create a connection in a health service that’s focused towards what the patient wants and we can start to collect data on that, then that would be really interesting for us.

B003

In the population health aim of health care, specific organizational factors were considered barriers to digital health care planning and implementation. The lack of adequate organizational digital health vision, strategy, or governance meant that health service improvements “can’t be made to fit in with the regulations that exist” (A003). Interoperability challenges were faced within services and across external health care provider communities, creating communication challenges for patient care and causing “a lot of frustration in the GP or primary care community in terms of them being able to access information from a tertiary centre” (B005). Digital systems at high-maturity sites have the capability to track a patient’s journey, leading to more efficient care delivery. Digital systems provide a detailed record of a patient’s history and allow providers to make decisions based on the most accurate and up-to-date information, giving “the manager a sort of 30,000 feet view...to make sure the care we provide is quite holistic in nature” (A007). Accurate and accessible clinical data helped to mitigate the clinical risks at high-maturity sites. Participants discussed risks including medication errors, clinical incidents, or patient harm:

We do have less incidents and less harm to our patients, because the digital system has helped that happen.

M004

For health care costs, the resource burden of investments required to establish and maintain digital maturity and to train the staff was noted. Training makes users more comfortable with the systems and teaches how to adjust the programs to their specific needs:

[T]here’s enormous amount of work...to actually train people up to use it but in terms of ongoing efficiency of use, I think that that’s lacking at the moment.

B004

For provider experiences in high-maturity sites, system usability was variable and change fatigue was reported as a negative impact:

Change fatigue, it’s quite a big burden on the clinical side and the nursing staff, and they seem to—the nursing staff seem to be the ones that has the biggest burden of the documentation and they seem to get the most changes because they’re using more of the system.

A002

#### Intermediate Digital Health Maturity

At sites classified as intermediate digital health maturity, telehealth was considered important for “unlocking that care closer to home” (C007). The patient experience was highly variable based on the digital literacy of the patient. One participant reported that poor patient digital literacy that negatively influence information accuracy could be harmful:

The concern would be if we hand over the responsibility of the care, the optimisation of the patient to the patient, will they engage with that, or will we end up chasing them rather than them meeting the milestones?...Would they actually give us the [required clinical] information? [It could] ultimately result in a day surgery cancellation.

E002

In addition, within the patient experience aim of health care, concerns about the decrease in patient-provider communication using digital technology were raised:

I think that has been a change to the way that we deliver care with that decreased communication and touch.

O003 and O009

Tracking patient experience data is more feasible as maturity increases, and participants appreciated the opportunity for further growth in “values-based health care” (O011). As health care systems digitally transform, patients are disadvantaged by the difficulty of finding and maintaining their own medical records. This was most evident in rural and remote areas.

In a similar way that telehealth improves flexibility for the *individual* patient (reported under the patient experience aim), it increases access to services for entire *populations* (reported in population health) that would otherwise be devoid of certain types of specialized medical care:

There is a lot better communication...The collaborative approach to health care provision is definitely something that is sold as part of the big successes...

P013, P010, and P011

Clinical risk mitigation was a benefit that began to be observed at intermediate-maturity sites. The ability to access and visualize accurate clinical data and risk improves health care quality and safety:

If I’ve got the data, if I’ve got good, solid data, then I can influence the way we design our hospitals, I can influence the cares that we’re providing to our patients.

E013

Interoperability issues were reported as an impediment to efficient population health:

Are we ever going to be able to see the data from these external agencies at all?

O012

Integration improvements would perceivably benefit care quality and patient safety:

We need to integrate all of hospital and health services to the local care providers, like GPs and outpatient community thing, which is still not very well integrated. I think that ought to make a massive difference to the patient outcomes, communication, early notification, improve morbidity and even mortality.

K004

Health care costs associated with investments in digital health were noted. Financial constraints may limit the potential positive impact of the EMR:

Anything we do technology wise...we tend to run out of money and fall down at the implementation stage and don’t always see the benefits of what we could do.

E005

The resource burden of developing a skilled digital health workforce was noted at intermediate-maturity sites. A workforce that is skilled at data analytics was desired:

[A lot of] data’s there. Do we have the ability to see it all? I don’t think so. I don’t think our data team is at that stage simply due to staffing numbers...And then do we have the smarts to create a dashboard out of that? I don’t think we’re close to that yet.

P017

The economic benefit of digital health investments was not clear as reported at intermediate-maturity sites. The complexity of conducting economic evaluations to track outcomes across the patient care journey inhibits this analysis, which could demonstrate value:

economic evaluation...was quite difficult...there needs to be a lot more transparency in data sharing...to make sure that we’re getting the best bang for our buck [sic] in terms of our targeted key performance indicators.

G005

For providers at intermediate-maturity sites, poor usability caused frustration among users. Usability issues stem from poor system design, inefficient data entry, and a lack of local customizations suitable for specific clinical tasks:

In terms of your user interface, it’s not like Apple, it’s more like PC. It’s not pretty...The usability side of it has been a point of disappointment for the clinicians...It certainly feels like you’re stepping back in time a little bit when you’re using it.

K002

The management of clinician workload through digital systems has emerged as a beneficial impact for the workforce at intermediate-maturity sites. The transparency of the staff workload was possible through tracking the digital data:

We end up getting reports and it gives us basically the hours per patient they required. It gives us an understanding of the workload within and the types of patients that we’re caring for at a ward level but at the service level and our [healthcare system] level.

C005

#### Low Digital Health Maturity

In the lowest-maturity sites, telehealth provided health care access and flexibility for patients “for convenience” [D009]; however, the patient experience was hindered by challenges in finding and maintaining patient health records in a “hybrid” paper and digital model. When multiple systems are used, the patient experience diminishes:

I received feedback recently where a consumer...who lived in a remote community...was quite confused about the fact that her records were paper based, and some were electronic. So, I think that sometimes that can be a little bit disjointed with things being on [digital system] and then paper-based documents, because people assume that if it’s not in the paper-based documents, it doesn’t exist.

J006

For population health, telehealth ensured service provision in rural and remote Queensland and enabled access to specialist services for those who previously preferred in-person consultations with “no disruption to the patient’s life” (D004). Tracking patient information across care settings was not observed as “multiple clinicians are being asked the same questions 5 or 6 times" (L004), inhibiting effective care delivery. Organizational culture change and clinical leadership in digital health were considered necessary:

That culture change piece within IT is one of the challenges I’ve continued to face. Having a clinician in charge of an IT shop is an interesting position. However, it is essential to have that if you’re going to be pushing that transformational change, that digital change.

H009

In terms of the health care cost aim of health care, the resource burden of investments required to establish and maintain digital maturity including ongoing hardware costs was recognized. The resource requirements to develop a skilled digital health workforce were considered burdensome.

For the provider experience, poor usability of systems was a negative impact, which lengthened tasks, leading to clinician frustration with the system, *“I was not a fan,...it doesn’t work well”* (L006). Clinicians emphasized their desire to be involved in the digital transformation journey from the beginning to ease the change fatigue:

Sometimes that puts a bad taste in people’s mouth, too, if you upset the long-standing workforce just to bring in—just for change. I think, if to do change, we’ve got to bring them on the journey with us and make them feel like they’re a part of the journey, instead of us just telling them that we’re going digital, and that’s what that—and if you don’t jump on board, well then tough bikkies [sic].

L008

In terms of provider acceptance at low-maturity sites, some staff members were hesitant to change to new systems. Poor infrastructure and network connectivity were mentioned at low-maturity sites, rendering digital solutions unusable for clinicians at times and requiring reversion to previous documentation systems:

We have an outdated version of that [system] which we can’t update because our server won’t support it,...the internet speed makes it essentially unusable on some days.

L007

#### Comparison of Perceived Impacts by Health Care System Digital Health Maturity

Health care providers working in sites with high digital health maturity reported more positive impacts than those working in with intermediate- and low-maturity sites. The population health aim had the highest number of positive impacts reported by health care staff at higher-maturity sites. Concerning the patient experience aim of health care, intermediate and high digital health maturity were associated with maintaining a patient health record and tracking patient experience data, while telehealth enabled access and flexibility across all digital health maturity categories. Negative impacts on patient-provider face-to-face communication were also reported at intermediate- and high-maturity sites. In terms of the population health aim of health care, patient journey tracking and clinical risk mitigation were reported as positive impacts at higher-maturity sites, and telehealth enabled health care access and efficiencies across all digital health maturity categories. Limited interoperability and organizational factors were universally negative impacts limiting effective health service delivery. With respect to health care costs, the resource burden of ongoing investments in digital health and a sustainable skilled workforce was reported, with the economic benefit of digital transformations being unclear at intermediate-maturity sites. As for the provider experience, the negative impacts of poor usability and change fatigue were universal, regardless of the digital health maturity. Acceptance was variable across low- and intermediate-maturity sites and was not an observed finding at high-maturity sites. Effective management of clinician workloads was observed in intermediate-maturity sites, but limited network and infrastructure negatively impacted provider experiences at low-maturity sites.

## Discussion

### Principal Findings

In a complex health care environment with various existing and emerging digital health interventions, digital maturity offers the most comprehensive measurement of digital excellence [11]. This is the first study to equate the digital health maturity of health care systems with outcomes at scale, addressing a critical research gap in evidencing the impacts and unanticipated consequences of digital health transformations [10]. Comparisons between the 3 digital maturity levels provide insights into the association between improved digital health maturity and better system outcomes.

### Key Insights

#### Overview

The perceived impacts of digital transformation varied across the quadruple aims for each digital maturity category: mostly negative for provider experiences, increasingly positive for patient experiences and population health, and largely unknown for health care costs. Insights from this research illustrate the various people, processes, and outcomes impacted by advancing digital health care in Queensland. In following sections, we focus on 3 key insights, comparing our findings with the existing evidence from the global literature.

#### Key Insight 1: Telehealth Had Positive Impact on the Patient Experience and Population Health Regardless of Digital Maturity

This study found that telehealth had a positive impact on both patient experience and population health across all levels of digital maturity. Despite the lowest-maturity sites lacking a full digital integration in health services, the expansion of telehealth driven by the COVID-19 pandemic had a positive impact. This positive sentiment is mirrored in the current evidence on telehealth in the COVID-19 era, with articles reporting a “celebratory sentiment about the use of telehealth” [36]. Telehealth is appreciated for its potential to overcome geographic, socioeconomic, cultural, and language barriers to give marginalized communities better access to essential health services [37]. Certainly, greater adoption in resource-limited settings and low- and middle-income countries has the potential to transform global population health [36]. In various randomized trials, video consultations were associated with high satisfaction among patients and staff and lower transaction costs while observing no differences in disease progression or service use when compared with traditional in-person care [38].

Positive perceptions of telehealth for patients and health care delivery in Queensland existed, irrespective of the COVID-19 pandemic. Even before the COVID-19 pandemic, Queensland was an early adopter of telehealth care models due to its geographically dispersed population. Although Australia observed an immediate increase in monthly telehealth care delivery after the onset of COVID-19 and associated changes to the telehealth funding model [39], rural and remote communities were already leveraging its benefits. Travel time savings and productivity gains are economic benefits of telehealth. For example, when each in-person consult was substituted with telehealth, a net travel time saving of 0 to 2.5 days was observed in addition to a societal productivity gain of approximately A$304 (US $207) in value [40]. With a peak in telehealth awareness driven by changes in models of care fueled by the pandemic, increasing opportunities for telehealth should ultimately help address patient needs and lead to sustainable health care services [41]. Findings from this study contribute to the positive influence of telehealth on health care provision, access, and flexibility, signifying advantages across the patient experience and population health quadrants regardless of digital health maturity.

#### Key Insight 2: Provider Experiences of Usability and Change Fatigue Are Negative Impacts of Digital Health Transformations

Experience and human factors are often overlooked in health care decision-making [19]. Disrupted ways of working and slower workflows introduced with EMR implementations have been shown to contribute to clinician frustration [42,43]. In this study, change fatigue was universal at all levels of digital maturity, and poor usability was more commonly reported in lower-maturity sites, with participants reporting issues associated with suboptimal user interfaces, navigational challenges, excessive training, and role fit. The absence of acceptance as a reported impact in high-maturity sites suggests that health care staff have adapted to digital health care delivery. Similarly, staff in high-maturity sites reported mixed sentiments on system usability in comparison to staff in intermediate- and low-maturity sites who reported poor usability as having a negative impact on the provider experience.

The usability concerns and change fatigue observed by the study participants correlate to the body of literature on clinician burnout, a syndrome of emotional exhaustion, depersonalization, and lowered personal accomplishment [44]. In the digital hospital literature, the following factors that exacerbate burnout have been identified: excessive documentation time, poor digital design that takes time away from patient care, and interaction with EMRs after work hours [45,46]. Outcomes of clinician burnout extend beyond individual psychological effects to reported poorer care delivery and lower patient satisfaction [18]. The Mayo Clinic reported that a higher physician-rated EMR usability score was associated with lower reported burnout [47]. With the ubiquity of digital health and increasingly concerning workforce challenges facing global health care systems, balancing the benefits of digital records (eg, safety, quality improvement, and risk management [48]) against provider satisfaction needs to be appropriately managed. Principles include the removal of extraneous cognitive load and efforts to align the health care system with the human, instead of the human with the health care system [19]. By measuring and addressing provider experiences as health care systems enhance their digital maturity, we can alter our practice and implementation in a way that accommodates the needs of our health care providers and ultimately improve all other health care aims.

#### Key Insight 3: Interoperability Needs to Be Addressed for Health Care Systems Undergoing Digital Transformations to Achieve Improved Population Health

Interoperability describes the data and information exchange between systems within organizations; across care settings; and with patients, caregivers, and families [3]. The participants reported negative experiences of poor interoperability across all levels of digital maturity. In high-maturity sites, however, enhanced patient journey tracking was reported, which suggests effective intraorganizational interoperability. Providers reporting negative experiences with system interoperability outlined challenges with information exchange, which inhibit coordinated care and create negative impacts on population health. Information exchange with external primary care providers was a particular concern, leading to feelings of hopelessness and frustration in terms of provider experience aim. In primary care settings, high levels of EMR interoperability have shown time-saving advantages for specific tasks including preparing laboratory reports, requesting laboratory orders, prescribing medications, and writing referrals [49]. Generally, interoperability contributes to EMR acceptance for physicians [50].

Successful interoperability relies on technical considerations (data types, structures, and standards) [51] and can be examined using interoperability-specific maturity models (eg, Health Information Systems Interoperability Maturity Toolkit) [52]. Comprehensive digital health capability assessments, such as the DHI used in this study [26], examine strengths and identify opportunities for health care providers to advance intraorganizational and extraorganizational information exchange in the interoperability dimension. Cross-nationally, the Global Digital Health Partnership is working to provide strategic direction to ensure that interoperability is enabled at a global scale, acknowledging the reality of variations in health care delivery systems, infrastructure, and participation [51]. Considerations for establishing best practices for interoperable EMRs include adopting international standards (eg, Fast Healthcare Interoperability Resources, Logical Observation Identifiers Names and Codes, and Systematized Nomenclature in Medicine Clinical Terms); addressing education and awareness; strengthening privacy knowledge; and exploring alternative solutions that do not require an entire retrofit to existing systems to deliver data [53].

### Implications

Our findings indicate that more digitally mature sites report better outcomes. Health services should aim to improve their digital health maturity to improve outcomes across the quadruple aims of health care. All health services should prioritize building a strong digital health strategy, address poor interoperability between systems, manage change fatigue, and improve system usability. Low digital maturity sites should incorporate education on digital patient-provider communication and embed patient experience tracking as they implement digital health. Telehealth offers the best opportunity for a positive impact, even in low-maturity organizations. Health organizations need to determine systematic approaches to valuing digital transformations that will help manage investment and workforce requirements. Health service decision makers should evidence the clinical benefits of advancing digital maturity, such as improved clinical risk mitigation, better patient journey tracking, and the ability to monitor patient experiences.

Overall, to ensure digital excellence, it is essential for health services to consider the impacts on patient experience, population health, health care costs, and provider experiences at various levels of digital maturity. Similarly, acknowledging the benefits and managing the disbenefits helps health care decision makers maximize the value of digital health to address the quadruple aim of health care, making it essential to plan a digital roadmap that delivers this value for all stakeholders. Digital maturity and digital maturity models in health care offer a path to evolution regarding the aspects under examination [54]. However, evidence to support the benefits of using them remains largely absent [14]. Practitioners and researchers will need to remain clear about the processual and organizational actions required for transformation, in addition to the technological actions [54]. Defining the local governance and policy requirements to effect change in these actions and deliver on the 3 implications of this study will be needed. The dimensions and subdimensions of existing [26] and aggregated models [9,10,14] offer an important starting point for consideration to identify focus areas and prioritize efforts.

### Recommendations for Future Research

Correlation studies exploring the association between increasing maturity and quantitatively measured health care performance outcomes (eg, mortality and hospital readmissions) would complement the perceived impacts reported in this study. Similar methodological approaches in different health care settings will be important to expand the evidence base. Digital transformations in health care are continuous, with system enhancements via periodic investment in software and hardware upgrades impacting function and usability. Thus, future research should explore the longitudinal perceptions of system transformation beyond the initial postimplementation phase. The health care cost aim has disproportionately fewer impacts reported, and the economic evaluation is a necessary and important area of future work, incorporating health economics and business research methodologies to strengthen the value proposition of advancing maturity.

### Limitations

Sites were grouped according to the quartiles of the DHI scores of the 16 health care systems, with a relatively small IQR of 116.75 to 166.75 on a 400-point scale. This method of grouping the sites delineated the respective digital maturity levels well, although the scores of high digital maturity sites ranged from 166.75 to a maximum score of 193, which, while indicative of high digital health maturity in Australia, is still low when compared with the maximum score of 400. This is reflective of the ongoing digital health transformation underway. The methodological limitations of the enlisted study design include participant sampling bias and the absence of patient or consumer experiences. Subjective accounts of the impacts reported in this study were not triangulated with the health service performance measures. In-person data collection was not possible owing to the COVID-19 pandemic restrictions, which may have elicited additional insights in addition to the web-based videoconference mode of data collection. The significant health workforce impacts of health care delivery during the COVID-19 pandemic period may have influenced participant reports in terms of their experiences, perspectives, and impacts.

### Conclusions

Using a mixed methods case study approach across the large Australian state of Queensland, the various impacts of digital transformation were examined and stratified according to the digital health maturity of the sites. The study results indicate that higher digital health maturity is associated with better outcomes. Higher maturity was associated with maintaining a patient health record, tracking patient experience data, tracking the patient journey, and mitigating the clinical risk. The negative impacts reported in low-maturity sites include variable provider acceptance, network and infrastructure issues, patient digital literacy concerns, and limited capability to find and maintain patient health records. The findings also indicate that telehealth has positive impacts on patients and population health regardless of health care system digital maturity; provider experiences of usability and change can be challenging, and interoperability needs to be addressed for health care systems undergoing digital transformations. When used as a strategic framework for digital health care improvement, the quadruple aim of health care focuses the transformation efforts on enhancing the patient experiences, improving population health, reducing the cost of care, and improving the provider experiences. Monitoring the various outcomes of advancing digital maturity helps organizations navigate digital decision-making to leverage the benefits of technology-enabled models of care while mitigating negative impacts that may threaten care quality.

## Appendix

Multimedia Appendix 1 Digital Health Indicators and dimension level scores for high, intermediate, and low digital health maturity sites.

Multimedia Appendix 2 Leximancer analysis—most frequent and relevant concepts.

Multimedia Appendix 3 Representative participant quotes for each maturity category and impact.

## Abbreviations

DHI: Digital Health Indicator
EMR: electronic medical record
HIMSS: Healthcare Information and Management Systems Society
