# Supplementary material for: Perceived Impact of Digital Health Maturity on Patient Experience, Population Health, Health Care Costs, and Provider Experience: Mixed Methods Case Study
Source: J Med Internet Res. 2023 Jul 18;25:e45868. doi: 10.2196/45868 (PMC10394505; doi:10.2196/45868)
Supplement: Multimedia Appendix 1 [file jmir_v25i1e45868_app1.docx]

**MULTIMEDIA APPENDIX 1: Digital Health Indicator (DHI) and dimension level scores for high, intermediate and low digital health maturity sites (sorted by ascending DHI score)**

| **Site** | **DHI dimension score (0-100)** | | | | **DHI score (0-400)*** | **Group** |
| --- | --- | --- | --- | --- | --- | --- |
|  | **Governance and Workforce** | **Interoperability** | **Person-Enabled Health** | **Predictive Analytics** |  |  |
| 2 | 19 | 27 | 23 | 21 | 78 | Low digital health maturity |
| 1 | 23 | 24 | 33 | 7 | 84 |  |
| 7 | 37 | 24 | 28 | 24 | 97 |  |
| 15 | 44 | 38 | 23 | 16 | 114 |  |
|  |  |  |  |  |  |  |
| 16 | 51 | 43 | 29 | 29 | 125 | Intermediate digital health maturity |
| 13 | 49 | 38 | 20 | 49 | 125 |  |
| 6 | 51 | 58 | 29 | 36 | 141 |  |
| 3 | 63 | 49 | 35 | 36 | 151 |  |
| 12 | 59 | 45 | 36 | 35 | 158 |  |
| 9 | 47 | 56 | 52 | 24 | 160 |  |
| 8 | 97 | 28 | 32 | 30 | 165 |  |
| 14 | 58 | 55 | 54 | 21 | 166 |  |
|  |  |  |  |  |  |  |
| 11 | 67 | 65 | 44 | 22 | 167 | High digital health maturity |
| 4 | 61 | 63 | 39 | 56 | 179 |  |
| 5 | 74 | 65 | 38 | 45 | 181 |  |
| 10 | 61 | 60 | 59 | 37 | 193 |  |
| * A proprietary algorithm is applied to calculate the total DHI score (i.e., the total score is not the sum of the dimension scores). | | | | | | |
