# Supplementary material for: Perceived Impact of Digital Health Maturity on Patient Experience, Population Health, Health Care Costs, and Provider Experience: Mixed Methods Case Study
Source: J Med Internet Res. 2023 Jul 18;25:e45868. doi: 10.2196/45868 (PMC10394505; doi:10.2196/45868)
Supplement: Multimedia Appendix 2 [file jmir_v25i1e45868_app2.docx]

**MULTIMEDIA APPENDIX 2: Leximancer analysis most frequent and relevant concepts**

**Group 1 (high maturity) top 25 most frequent and relevant concepts**

| **Concept** | **Count** | **Relevance** |
| --- | --- | --- |
| patient | 446 | 100% |
| system | 340 | 76% |
| care | 321 | 72% |
| patients | 320 | 72% |
| time | 311 | 70% |
| health | 303 | 68% |
| data | 296 | 66% |
| people | 296 | 66% |
| information | 289 | 65% |
| need | 285 | 64% |
| use | 273 | 61% |
| work | 258 | 58% |
| digital | 246 | 55% |
| able | 239 | 54% |
| clinical | 230 | 52% |
| look | 228 | 51% |
| doing | 220 | 49% |
| hospital | 180 | 40% |
| different | 177 | 40% |
| service | 167 | 37% |
| looking | 166 | 37% |
| guess | 161 | 36% |
| team | 159 | 36% |
| systems | 155 | 35% |
| terms | 147 | 33% |

**Group 2 (intermediate maturity) top 25 most frequent and relevant concepts**

| **Concept** | **Count** | **Relevance** |
| --- | --- | --- |
| health | 711 | 100% |
| patient | 678 | 95% |
| people | 639 | 90% |
| data | 591 | 83% |
| time | 558 | 78% |
| need | 550 | 77% |
| patients | 525 | 74% |
| use | 495 | 70% |
| system | 492 | 69% |
| care | 490 | 69% |
| digital | 487 | 68% |
| information | 484 | 68% |
| look | 433 | 61% |
| doing | 421 | 59% |
| work | 407 | 57% |
| hospital | 372 | 52% |
| clinical | 369 | 52% |
| able | 354 | 50% |
| service | 348 | 49% |
| different | 325 | 46% |
| team | 320 | 45% |
| staff | 310 | 44% |
| guess | 292 | 41% |
| having | 275 | 39% |
| down | 267 | 38% |

**Group 3 (low maturity) top 25 most frequent and relevant concepts**

| **Concept** | **Count** | **Relevance** |
| --- | --- | --- |
| need | 251 | 82% |
| use | 245 | 80% |
| health | 219 | 72% |
| digital | 215 | 70% |
| patient | 208 | 68% |
| system | 198 | 65% |
| patients | 189 | 62% |
| care | 186 | 61% |
| time | 185 | 60% |
| able | 178 | 58% |
| information | 177 | 58% |
| look | 164 | 54% |
| doing | 159 | 52% |
| data | 155 | 51% |
| service | 155 | 51% |
| work | 147 | 48% |
| systems | 127 | 42% |
| hospital | 127 | 42% |
| down | 116 | 38% |
| different | 114 | 37% |
| staff | 111 | 36% |
| support | 102 | 33% |
| better | 100 | 33% |
| trying | 92 | 30% |
| day | 92 | 30% |
