# Supplementary material for: Perceived Impact of Digital Health Maturity on Patient Experience, Population Health, Health Care Costs, and Provider Experience: Mixed Methods Case Study
Source: J Med Internet Res. 2023 Jul 18;25:e45868. doi: 10.2196/45868 (PMC10394505; doi:10.2196/45868)
Supplement: Multimedia Appendix 3 [file jmir_v25i1e45868_app3.docx]

**MULTIMEDIA APPENDIX 3: Representative participant quotes for each maturity category and impact**

| Quadruple aim of healthcare impact | High Maturity | Intermediate Maturity | Low Maturity |
| --- | --- | --- | --- |
| **Patient experience** | | | |
| Telehealth for healthcare access and flexibility | Positive: *“There’s certainly a younger generation that are more connected, both from an internet perspective and obviously from a kind of getting it perspective, when it means they don’t have to get in their car and drive for such long distances, or get on a plane, and stay overnight with all of the things that are encompassed in that - making sure your kids are being looked after, or your grandparents are being kept alive.” - A10* | Positive: *“So telehealth was the key I suppose, that was still the bullet for us to unlocking that care closer to home” – C007* | Positive: *“They did implement…a really simple system. …They were able to link with people directly on their iPads at home. And they did it for convenience, but what they then also found was that kids behaved much more like themselves when they were in their own home. In the clinic they wouldn’t do stuff, they wouldn’t show – you couldn’t assess their development because they wouldn’t get out of the chair.”* - D009 |
| Patient-provider communication | Negative: *“There's been a lot of feedback, which is around patients feeling that the computer or the digital system sits between them and the nurse, or them and the doctor or the attention of the nurse or the doctor or the allied health professional, is on the computer, rather than on the patient.” - F009&10* | Negative: *“One thing I’ve noticed …is that with the use of digitalised obs machines, …there is that decreased communication and touch with the patient. … once upon a time, you would touch them, you feel them when you put the blood pressure cuff on. Now we just put the cuff on and leave it and come and press a button and take their obs regularly. I think that has been a change to the way that we deliver care with that decreased communication and touch.”* *– O003 and O009* | N/A |
| Patient digital literacy | N/A | Negative: *“The concern would be if we hand over the responsibility of the care, the optimisation of the patient to the patient, will they engage with that, or will we end up chasing them rather than them meeting the milestones? ….Would they actually give us the [required clinical] information? [It could] ultimately result in a day surgery cancellation.” – E002* | Mixed: *“Younger people are more open to discussion about the use of apps for tracking different parameters, they’re more open to the use of anything you can fit on a smartphone. Older patients, it can be a little more difficult, although we have an older patient at the moment who is profoundly deaf who uses his electronic translator on the ward and in consultations with great effect.” - L006* |
| Patient experience data tracking | Positive: *“We’re starting to play with this idea of if we can create a connection in a health service that’s focused towards what the patient wants and we can start to collect data on that, then that would be really interesting for us.” - B003* | Negative: *“I think we’re very misguided in the way we capture data, and using our data analytics, because we’re so focused on a business... rather than the actual outcomes and the values of what we do. What we are so busy doing when we’re collecting our data is that we’re reporting on activities. So, has the nurse seen the patient and done a care plan? Tick! Where’s the value in that and where’s the outcome of that? How many people has the physio seen today in their schedule? So what? It doesn’t mean anything to the patient, you’ve got to measure things differently. I think we’ve got to sway to values-based healthcare and then have a system that supports it.” – O011* | N/A |
| Find and maintain patient health record | N/A | Mixed: *“I also like the record keeping, there’s so much more information for the patient. I think there’s - from a patient [use] perspective, I think ieMR has a lot more potential [which is not yet realised].” – K003* | Negative: *“I received feedback recently where a consumer …– who lived in a remote community – …was quite confused about the fact that her records were paper based, and some were electronic. So, I think that sometimes that can be a little bit disjointed with things being on [digital system] and then paper-based documents, because people assume that if it's not in the paper-based documents, it doesn't exist.”*  *– J006* |
| **Population health** | | | |
| Telehealth for healthcare access and efficiency | Positive: “*Telephone and particularly a video conference medium to provide health care services. I think one of the things that we have done well here is actually invested workflows and processes to sustain that.”- B10* | Positive: “*There is a lot better communication with GPs [due to telehealth]. The collaborative approach to healthcare provision is definitely something that is sold as part of the big successes of it as well.” – P013, P010, P011* | Positive: *“Particularly after COVID, I mean we haven’t been able to travel very much. So it’s so important out here [in the country] …the uptake has been quite significant over that period of time. … So a lot of specialists are now using – they wouldn’t before they’d want people to come down face to face. It’s a lot of money, it’s a lot of travel, it’s a lot of time for these people. So now we just sit in the back office on a nice comfy chair and have a Telehealth review with the specialist and there’s no disruption to the patient’s life.” - D004* |
| Operational barriers [strategy, policy, vision] for health service delivery | Negative: *“Because the public health system is so glorious and so regulated they can’t actually use a system that is actually effective because it’s illegal so therefore they have no communication or very poor communication. They’re not utilising the electronic services that are available because they’re either too expensive or they can’t be made to fit in with the regulations that exist.” – A003* | Negative: *“If we were able to put that information in the hands of clinical teams, and particularly clinical leaders, who could then do sort of continuous performance improvement with the information that they’ve got, we would unleash a lot of capability and capacity within the organisation to improve it from within.’ – K002* | Negative: *“That culture change piece within IT is one of the challenges I’ve continued to face. Having a clinician in charge of an IT shop is an interesting position. However, it is essential to have that if you’re going to be pushing that transformational change, that digital change.”* -*H009* |
| Interoperability between systems | Negative: “*And you just go, “Well, that also doesn’t work very well, because at one point that information will then need to be supplemented or enriched by another data set somewhere.” And so, you’ve a number of these systems that work really, really well in isolation, for their specific thing, but we live in a complex healthcare system.” – F006/7* | Negative: “*Are we ever going to be able to see the data from these external agencies at all?’ – O012*  *“We need to integrate all of hospital and health services to the local care providers, like GPs and outpatient community thing, which is still not very well integrated. I think that ought to make a massive difference to the patient outcomes, communication, early notification, improve morbidity and even mortality.”* – K004 | Negative: “*Day-to-day, our management, we have our charts, that someone will come in and we have our QAD tools that we use and document on paper, and then we have a medical director that we’re meant to use as well. I’ve used ieMR, and I don’t know why we don’t have that sort of electronic records out here, I’m miffed by it. Because the use in between all the health services, and people being required to travel a lot in between the hospitals, it would make sense to me.” – D005* |
| Tracking patient journey | Positive: *“It gives the manager a sort of 30,000 feet view of how their patient is tracking in terms of their overall risk assessment, and the other mandatory assessment that we need to complete, as a part of their journey, to make sure the care we provide is quite holistic in nature.” - A007* | Mixed: *“The best thing is the Viewer and My Health Record – they're the best things that we have. Because we’re not flying blind on people's histories anymore. You know if they've been to another hospital.” – C002*  “*We don't have the digital platform in our health service. We're on the paper based. So scanning notes in and sending them to receiving facilities is a challenge. When you're transferring patients to tertiary facilities, [records] have to be photocopied and then sometimes when you're receiving patients back from tertiary facilities, they'll say, "It's in the records." "Well, actually no, can you just print those out for us." It has that implication at the other end as well, that we're not with the digital world.’ – E009* | Negative: *"I find nursing is behind in that world we have, while we have the latest infusion pumps and ECG monitors and all those sort of things, it's that use of electronic medical records and data precision tools that I think needs a significant uplift for everybody- medical, nursing and allied health. Patients are still seeing multiple clinicians and are being asked the same questions five or six times, which is extremely frustrating for them, whereas if we had an electronic tool, the information would have only been asked once, and it'd already be accessible to every clinician.” – L004* |
| Clinical risk mitigation | Positive: *“We do have less incidents and less harm to our patients, because the digital system has helped that happen.’- M04* | Positive: “*If I’ve got the data, if I’ve got good, solid data, then I can influence the way we design our hospitals, I can influence the cares that we’re providing to our patients, I can influence the fact that we’re going to have zero pressure injury rate and as an educator, …it’s really important that you build solid social capital. If you have good data, that lends itself to you being somebody who’s viewed in a positive fashion so then you can influence change”* – E013 | N/A |
| **Healthcare costs** | | | |
| Resource burden of ongoing investments | Mixed: *“The financial position that the state seems to be in every year in regards to, ‘We’ve got no money, make your efficiency savings.’ But you just need to balance that with giving staff the ability to actually use the technology effectively and efficiently to be able to deliver the patient care at the end of the day.” – M01* | Negative: *“Speaking generally, anything we do technology wise in [organization] we tend to run out of money and fall down at the implementation stage and don't always see the benefits of what we could do. Or ieMR is probably a good example where people go in thinking that the system will do something and then once we get to actually trying it or implementing it we realise the system isn't as good or doesn't quite do it the way we expected it would be done. That's the challenge.” – E005* | Negative: *“[Level 2 ieMR] that's probably $20 million and a lot of hard work and tears and two to three years to transition from what we have now into such a system, and unfortunately, we have missed the bus when [the organisation] did that with the other bigger site and referral hospitals, and we basically suffer from missing out with that up to now. … I think majority is lack of funds and the additional staff…” – J007* |
| Resource burden of a skilled workforce | Mixed: *“We're still learning about how to use electronic records in many ways and …that's one of the problems from my perspective is that sort of training and education around how to use it efficiently and effectively and through a quality and safety perspective is absent. So there's enormous amount of work in terms of when the system was implemented to actually train people up to use it but in terms of ongoing efficiency of use, I think that that's lacking at the moment.” - B004* | Negative: *“It knows when someone’s given antibiotics – prescribed antibiotics and given antibiotics, because it’s all done through the same system. So, all those timestamps exist. That data’s there. Do we have the ability to see it all? I don’t think so. I don’t think our data team’s at that stage simply due to staffing numbers. There’s three of them looking at that data and that’s a fairly hefty game for them. And then do we have the smarts to create a dashboard out of that? I don’t think we’re close to that yet.” – P017* | Negative: *“The challenge then is the upkeep and having the right people on the ground to be able to do that, and that’s where I think it will always fall short, because not everybody wants to live out bush. The super gurus, the super experts, the people with the skills.” – L009* |
| Economic benefit visible | N/A | Negative: *“economic evaluation … was quite difficult. … there needs to be a lot more transparency in data sharing … to make sure that we’re getting the best bang for our buck [sic] in terms of our targeted key performance indicators”* *– G005* | N/A |
| **Provider experience** | | | |
| Usability | Mixed: *“Because I think the first steps when you roll the ieMR out was the clinicians that were still just working through, “What’s the minimum number of clicks and entry points I need to make to get my job done?” As opposed to thinking about it and saying, “Well, actually, how am I absolutely going to use the system to the best benefit of all users of the electronic medical record including patients?” – B010* | Negative: *“In terms of your user interface, it’s not like Apple, it’s more like PC. So it’s not pretty. You need to know it quite well to be able to use it effectively. So I think the usability side of it has been a point of disappointment for the clinicians. …It certainly feels like you’re stepping back in time a little bit when you’re using it.”* *– K002* | Negative: *“We resisted the change to go to a full electronic pathology system, and so we still use [system] at the moment, we don’t use [system]. We print our pathology because we still have a paper-based chart, and the paper-based pathology is seen each day by the group. … I was not a fan of [system]. It doesn’t work well for group, it’s designed for individual clinician work.” – L006* |
| Change fatigue | Negative: *“Change fatigue, it’s quite a big burden on the clinical side and the nursing staff, and they seem to – the nursing staff seem to be the ones that has the biggest burden of the documentation and they seem to get the most changes because they’re using more of the system.” -A002* | Negative: *”Part of the issue is that the HHS does not do collaboration or engagement well at all. It’s really hard to get the value put in that space which is actually the main thing you need. That is the main thing you need for sustained change, and to get people on board and to reduce that resistance. So, that I think is my key, is actually walking them through it, doing it together, trouble shooting and being present.’ – O011* | Negative: *“Sometimes that puts a bad taste in people's mouth, too, if you upset the long-standing workforce just to bring in - just for change. I think, if to do change, we've got to bring them on the journey with us and make them feel like they're a part of the journey, instead of us just telling them that we're going digital, and that's what that - and if you don't jump on board, well then tough bikkies [sic].”* *– L008* |
| Acceptance | N/A | Mixed: *“The older the nurse, but I don't want to stereotype it, but yes, we do have some people that have been nursing for quite some time, and they voice their concerns about transitioning to a digital healthcare system.”-* C004 | Mixed: *“The Electronic Medical Record is a complete disaster … The disadvantages are the enumerate number of errors that can occur with the system. If I was doing it here in [hospital] we still have a mainly paper-based record, I could write the bloody thing out myself.”*  *– L006* |
| Management of clinician workloads | N/A | Positive: *“So each shift they go in [to the patient acuity system] and document how much care they provided for each patient under their care. We end up getting reports and it gives us basically the hours per patient they required. It gives us an understanding of the workload within and the types of patients that we're caring for at a ward level but at the service level and our [healthcare system] level.” – C005* | N/A |
| Network and infrastructure impacts | N/A | N/A | Negative: *“At the clinic we run [program] as the clinic software. We have an outdated version of that which we can’t update because our server won’t support it, … the internet speed makes it essentially unusable on some days.” – L007* |
